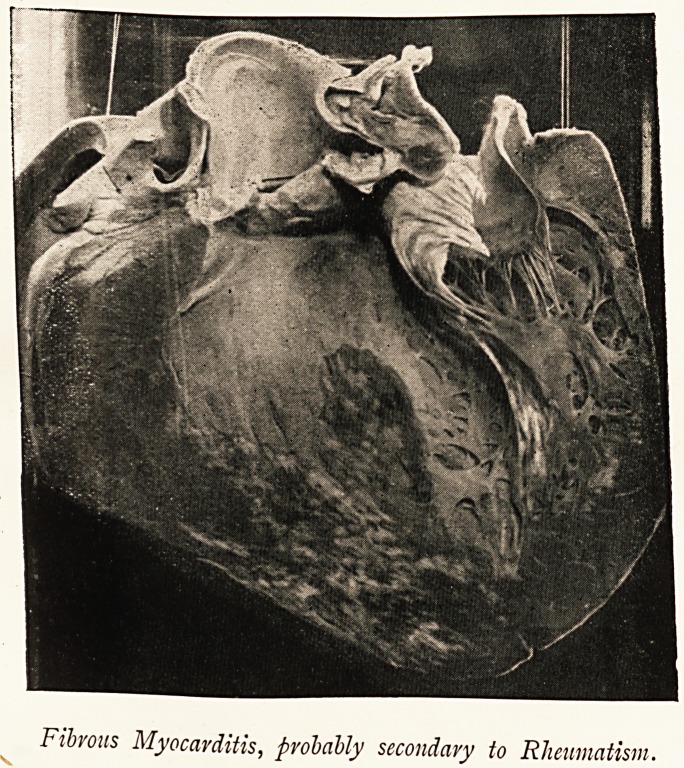# Rheumatic Disease of the Cardiac Muscle

**Published:** 1900-03

**Authors:** Theodore Fisher

**Affiliations:** Pathologist to the Bristol Royal Infirmary, Physician to Out-Patients to the Bristol Royal Hospital for Sick Children and Women


### RHEUMATIC DISEASE OF THE CARDIAC MUSCLE.

BY

THEODORE FISHER, M.D.Lond., M.R.C.P.,

*Pathologist to the Bristol Royal Infirmary, Physician to Out-Patients to the Bristol Royal Hospital for Sick Children and Women.*

---

ALL text-books on medicine speak of rheumatism as one of the causes of myocarditis, but the simple statement usually stands alone without comment. Clinically, the possibility of lesions of the cardiac muscle following rheumatism is rarely thought of, and this is not to be wondered at, since, apart from associated valvular disease or adhesion of the pericardium, their presence is rare. To the pathologist, fibrosis of the heart muscle complicating old rheumatic valvular disease is possibly of more interest than to the clinician; yet if it were possible to diagnose its presence the reverse would be the case, since the condition of the myocardium is of great importance when the question of prognosis is being considered. Small fibroid patches are frequently seen scattered through the heart muscle in fatal cases of mitral stenosis, and although I have not seen sufficient cases to justify any dogmatic statement upon the point, it seems to me very probable that a careful study of the heart muscle in cases of mitral stenosis would explain the strange variability in the length of life they present. Sometimes extreme stenosis may be met with in the *post-mortem* room at the age of sixty years, while cases of comparatively slight constriction will end fatally before the age of thirty. In both instances the valvular disease will probably have dated from an attack of acute rheumatism occurring before the age of twenty.

As already mentioned, some fibrosis of the cardiac muscle is not infrequently seen in the *post-mortem* room in cases of rheumatic valvular disease, but it has not been my experience to meet with an instance in which the interstitial changes have been better marked than in the case from which the accompanying illustration was taken.

A male patient, aged eighteen years, who had been attending the out-patient department under Dr. Edgeworth for aortic valvular disease was brought to the Bristol Royal Infirmary, having died suddenly while at work. At the necropsy the heart was found enlarged, weighing 21 oz. There was old thickening of the segments of the aortic valve, but the interest of the case centred in the condition of the heart muscle. Everywhere the inner surface of the left ventricle was thickly dotted with greyish-white spots of irregular shape, few of which were more than one-fifth of an inch across. Section of the heart wall showed these fibroid areas to extend through to the pericardial surface and to be rather more numerous in the neighbourhood of the apex than elsewhere. There was no evidence of there having been a previous attack of pericarditis, and the coronary arteries were healthy. Although I have been unable to ascertain whether the patient had suffered from acute rheumatism, the existence of an attack of that disease at some former period of his life may with safety be inferred. Old disease of the aortic valve found at the age of eighteen years can scarcely have any other cause. Possibly it may be doubted whether the fibrosis of the heart muscle should be attributed also to the rheumatic attack, and a syphilitic origin be suggested. Syphilitic disease of the heart, however, occurs late in the disease, and although multiple gummata have been described, I am not aware that any case of diffuse fibrosis of the heart has with good reason been assigned to syphilis.[1]

Curiously enough, there has recently been another case in the *post-mortem* room of the Bristol Royal Infirmary where fibroid disease of the cardiac muscle was associated with disease of the aortic valve at a much earlier period of life. In a child aged 4½ months, admitted under the care of Dr. Watson Williams for broncho-pneumonia, the heart was found after death to present thickening of the segments of the aortic valve, which were adherent, producing stenosis. In addition to this disease of the aortic valve, one of the musculi papillares was half converted into fibrous tissue, and microscopic sections of the heart muscle showed extensive recent increase of the interstitial tissue. The pericardium in this case, as in the former, was quite healthy. Here, again, there is no other cause to which the disease of the aortic valve can be assigned but that of rheumatism, and it is reasonable to consider that the disease of the cardiac muscle owed its existence to the same cause.

It has already been more than once mentioned that fibroid patches of small size are not uncommonly seen in the cardiac

[1] Recently we have had in the *post-mortem* room of the Bristol Royal Infirmary a case in which diffuse fibrosis of the cardiac muscle was associated with unmistakable evidence of syphilis in other organs. There was, however, aortitis immediately above the aortic valve, which had reduced the orifices of the coronary arteries to the size of pinholes, and the fibroid changes were no doubt in a great measure dependent upon interference with the blood-supply of the cardiac muscle.

*Fibrous Myocarditis, probably secondary to Rheumatism.*

muscle in fatal cases of mitral stenosis. Although we cannot ignore the view which attributes the morbid appearances to a disordered circulation of the heart wall, it is also reasonable to consider that the fibrous tissue indicates the former presence of myocarditis set up by an attack of rheumatism. In support of such a view is the fact that occasionally acute myocarditis may supervene in a case of old valvular disease. For example, a boy twelve years of age was admitted into the Bristol General Hospital, under the care of Dr. Harrison, for mitral stenosis. After being in the hospital for a week or two, he one day complained of a pain over the heart. Pericarditis was thought of, but on examination no rub could be detected. The cardiac action became rapid and feeble, and the following day the radial pulse could not be felt. The boy became drowsy, but continued restless, and frequent vomiting occurred. Four days after the first complaint of cardiac pain he died. In the absence of Dr. Michell Clarke, I made the *post-mortem* examination. The appearance of the heart was peculiar, and was so striking that it was at first commented upon by a member of the staff who was standing some distance away. The pericardium was perfectly healthy, but the ventricles were thickly dotted with small yellowish-white spots and streaks, the long axes of which ran in the direction of the muscle fibres. Not many spots were seen on the endocardial surface of the heart, but they thickly studded the cardiac muscle for some distance beneath the pericardium. The flaps of the mitral valve and chordæ tendineæ were thickened and the mitral orifice was stenosed. Microscopical examination of the heart muscle showed that the diseased condition was mainly due to degeneration of the muscle fibres, but some inflammation of the interstitial tissue was present. In this case acute disease of the myocardium was superadded to old disease of the mitral valve, and the myocarditis may with reason be attributed to a fresh attack of rheumatism affecting the heart. At least, precisely the same appearance may be seen in the hearts of children dying of rheumatic pericarditis, especially in cases that are not rapidly fatal. For example, in a boy aged seven years, who died in the Infirmary this year four months after the onset of acute rheumatism, which was complicated by pericarditis, the walls of the ventricles were everywhere studded with yellowish-white opaque streaks and small opaque dots.

It does not, however, require this visual evidence of disease of the heart muscle in cases of pericarditis to show us that there is poisoning of the cardiac walls. The rapid fatality of many cases is the best evidence of the poisoning. It cannot be considered possible that mere inflammation of the serous covering of the heart can arrest its action. But even if we consider the fatal event to be the result of the local inflammation when the whole of the pericardium is affected, cases occur in which the pericarditis is so limited that such an explanation cannot be held to be satisfactory. I have seen pericarditis rapidly fatal with great dyspnœa, where after death the inflammation of the pericardium was found to be limited to the surface of the right auricle. In rarer cases, where death occurs from cardiac failure shortly after an attack of rheumatism, without pericarditis or serious valvular lesion being present, we have the direct action of toxins upon the cardiac muscle still more strongly suggested. For example, a boy eight years of age was admitted into the Royal Hospital for Children and Women, Waterloo Bridge Road, who for two years had suffered from symptoms pointing to disease of the heart, which symptoms had become much aggravated a few weeks before admission, at the same time that pain and swelling of the knees had been present. On admission there was great dyspnœa and a rapidly-beating heart, the pulse being 140 to the minute. The dyspnœa became worse and the pulse more rapid, and at the end of ten days the boy died. After death not the least trace of pericarditis could be found. A previous attack of rheumatism was indicated by thickening of the aortic segments and of the cusps of the mitral valve. The aortic valve, however, was competent, and neither the thickening of this valve nor of the mitral could in any way account for death. There was evidence that the recent attack of rheumatism had affected the heart in the presence of very small recent vegetations on the segments of the aortic valve, and on microscopic examination the heart muscle showed remarkable injection of the blood-vessels, with slight inflammation of the interstitial tissue.

The only reasonable explanation for death seemed to be that the attack of rheumatism had caused cardiac failure without giving rise to pericarditis. This failure may with reason be looked upon as evidence of acute rheumatic poisoning of the heart muscle.

Instances where this poisoning is of milder character are probably not uncommon. In May, 1896, I read a paper before the Bristol Medico-Chirurgical Society, entitled "Is Mitral Regurgitation from Valvular Disease Common or Serious?" in which it was suggested that too great importance had been attached to lesions of the mitral valve in rheumatism, and that the possible effect of rheumatic toxins in weakening the cardiac muscle had been overlooked. The paper was published in the *Lancet*,[1] and elicited a letter from Dr. Lees,[2] in which he stated that he had noticed dilatation of the heart to occur frequently in cases of acute rheumatism. Dr. Lees,[2] in conjunction with Dr. Poynton, has since published his observations. From the point of view of prognosis, the question is an important one. If dilatation of the heart occurs commonly in acute rheumatism, then the mitral regurgitant murmur developing during the course of the disease will frequently not indicate a lesion of mitral valve, but be secondary to the cardiac dilatation.

Clinical experience seems to indicate that rheumatic poisoning of the heart may manifest itself in other ways. Dilatation and signs of valvular disease may be absent, yet there may be indications of weakening of the heart. After an attack of rheumatism, for example, a child may be noticed to have lost its activity, and will probably occasionally complain of pain over the cardiac area. Since the case is fresh in my mind, I may refer to an instance where such weakening followed chorea, which is so closely allied to rheumatism, and in children plays a part little inferior in the production of heart disease. A short time ago a boy aged nine years was brought to the out-patient department of the Bristol Children's Hospital by his mother, who said that he had never been well since an attack of chorea which occurred two years before. She described

[1] *Lancet*, 1896, ii. 170. [2] *Ibid.*, 273.

[2] *Brit. M. J.*, 1898, ii. 1129; *Med.-Chir. Tr.*, 1898, lxxxi. 401, 419.

him as having been "so bright" before, but now he was "so dilatory." On examination, the boy's heart appeared perfectly normal, but he complained of occasional pain below and outside the cardiac area. The pain occurred, he said, when he ran "a bit fast," after which he had to walk "a bit slow." Similar weakness following rheumatism is illustrated by such a case as the following: A girl aged eight years was brought to the Bristol Hospital for Sick Children on account of attacks of faintness. She had been a robust child until attacked by rheumatic fever four years previously, but had never been well since that illness. She was obliged to sit down when at play, and would turn pale when standing in her class at school. The symptoms obviously pointed to cardiac weakness, yet nothing abnormal could be detected on examination of the heart.

Occasionally some degree of tachycardia may apparently be due to rheumatism. For example, a girl aged four years was brought to the Bristol Children's Hospital in August, 1896, by her mother, who stated that the child often turned pale, complained of pain over the region of the heart, and that on putting her hand to the chest the mother could feel the heart beating forcibly and at a great rate. Physical examination of the heart revealed nothing noteworthy. It was, however, beating rapidly —148 to the minute. There was a history of pain and swelling in the joints, and, curiously enough, after the first visit to the hospital the child had to be kept in bed a week owing to pain and swelling in the right knee. This child has been seen again recently, and although the heart on physical examination still presents nothing noteworthy, the attacks of pallor, with rapidity of the pulse, are said to continue. In another case, in a boy aged eight years, the heart was said to beat at times "as fast as it could beat." The pulse was 132 to the minute in the out-patient room. Six months previously he had suffered from rheumatism, but the rapid beating of the heart had been noticed before the attack. The history of one attack of rheumatism, however, gives reason for suspicion that another had occurred earlier.

In young adults mild anginal attacks may date their origin from recovery from acute rheumatism, and in the absence of the evidence of any physical disease of the heart the attacks are generally diagnosed as hysterical. A neurotic disposition may undoubtedly aggravate the symptoms produced by cardiac distress, but because there is evidence of neurosis it does not follow that the whole disease is functional. Just as true epilepsy may be masked by hysterical fits, so I have little doubt that some cardiac weakness may lie unsuspected beneath attacks of hysterical angina pectoris. A few months ago I saw a young lady who suffered from mild anginal attacks following acute rheumatism. They had been diagnosed as hysterical, and she received little sympathy from her friends in consequence. Yet I could not doubt from the general symptoms that in this case there had been definite weakening of the heart.

The subject of the milder effects of rheumatism upon the heart is interesting and important. In a child it is easy to mistake physical weakness for laziness, and in a young female adult the symptoms produced by the same weakness may be thought evidence of hysteria. In either case injudicious advice may result in cruelty, if not in actual harm, to the sufferer.

## Figures and Tables

**Figure f1:**